# Effect of endurance exercise on microRNAs in myositis skeletal muscle—A randomized controlled study

**DOI:** 10.1371/journal.pone.0183292

**Published:** 2017-08-22

**Authors:** Jessica F. Boehler, Marshall W. Hogarth, Matthew D. Barberio, James S. Novak, Svetlana Ghimbovschi, Kristy J. Brown, Li Alemo Munters, Ingela Loell, Yi-Wen Chen, Heather Gordish-Dressman, Helene Alexanderson, Ingrid E. Lundberg, Kanneboyina Nagaraju

**Affiliations:** 1 Research Center for Genetic Medicine, Children’s National Health System, Washington, D.C., United States of America; 2 Department of Integrative Systems Biology, Institute for Biomedical Sciences, The George Washington University, Washington, D.C., United States of America; 3 Rheumatology Unit, Department of Medicine, Solna, Karolinska Institutet, Karolinska University Hospital, Stockholm, Sweden; 4 Departement of Pharmaceuticals Sciences, School of Pharmacy and Pharmaceuticals Sciences, Binghamton University, Binghamton, New York, United States of America; Universita degli Studi di Roma La Sapienza, ITALY

## Abstract

**Objective:**

To identify changes in skeletal muscle microRNA expression after endurance exercise and associate the identified microRNAs with mRNA and protein expression to disease-specific pathways in polymyositis (PM) and dermatomyositis (DM) patients.

**Methods:**

Following a parallel clinical trial design, patients with probable PM or DM, exercising less than once a week, and on stable medication for at least one month were randomized into two groups at Karolinska University Hospital: a 12-week endurance exercise group (n = 12) or a non-exercised control group (n = 11). Using an Affymetrix microarray, microRNA expression was determined in paired muscle biopsies taken before and after the exercise intervention from 3 patients in each group. Ingenuity pathway analysis with a microRNA target filter was used to identify microRNA transcript targets. These targets were investigated at the mRNA (microarray) and protein (mass spectrometry) levels in patients.

**Results:**

Endurance exercise altered 39 microRNAs. The microRNAs with increased expression were predicted to target transcripts involved in inflammatory processes, metabolism, and muscle atrophy. Further, these target transcripts had an associated decrease in mRNA expression in exercised patients. In particular, a decrease in the NF-κB regulator *IKBKB* was associated with an increase in its target microRNA (miR-196b). At the protein level, there was an increase in mitochondrial proteins (AK3, HIBADH), which were associated with a decrease in microRNAs that were predicted to regulate their expression.

**Conclusion:**

Improvement in disease phenotype after exercise is associated with increasing microRNAs that target and downregulate immune processes at the transcript level, as well as decreasing microRNAs that target and upregulate mitochondrial content at the protein level. Therefore, microRNAs may improve disease by decreasing immune responses and increasing mitochondrial biogenesis.

**Trial registration:**

ClinicalTrials.gov NCT01184625

## Introduction

Inflammatory myopathies, collectively known as myositis, are a group of heterogeneous rheumatic disorders characterized by chronic muscle weakness and fatigue [1, 2]. The pathology is characterized by extensive inflammation but also includes signs of skeletal muscle damage such as central nucleation, variation in fiber size, fibrosis, and fiber atrophy. Although its underlying cause is currently unknown, myositis is thought to be autoimmune in origin, given the presence of autoantibodies in more than 50% of patients [3]. Since these disorders involve both muscle and immune responses, anti-inflammatory and immunosuppressive drugs are currently used for treatment. In particular, glucocorticoids are an effective treatment because they target both the muscles and the immune system [4]. However, they have substantial side effects, such as muscle fiber atrophy, that can lead to further muscle damage when taken in large doses or for extended periods of time [5].

During the past decade, exercise has been recognized as an effective anti-inflammatory intervention in many chronic diseases [6]. It is well known that both skeletal muscle and immune pathways are altered by exercise [7]; therefore, exercise as a therapy is particularly relevant to myositis. Recent studies have shown that both resistance and aerobic exercise are well tolerated by polymyositis (PM) as well as dermatomyositis (DM) patients and may be used as a therapeutic intervention to improve disease outcomes. Resistance exercise training for 7 weeks has been shown to increase muscle strength, decrease levels of serum creatine kinase, and improve myositis intention-to-treat activity index (MITAX) scores when compared to baseline measurements [8]. At the molecular level, global gene expression microarrays show reductions in pro-inflammatory and profibrotic gene networks [9]; however, these changes are not observed at the histological level, in part because the inflammation does not occur evenly throughout myositis muscle and because there is a poor correlation between the degree of inflammation and the severity of symptoms [8, 10].

In addition to resistance exercise, endurance exercise training for 12 weeks has also been shown to provide functional benefit. Exercised patients have reduced disease activity, according to the International Myositis Assessment and Clinical Studies Group (IMACS) score, as well as increases in aerobic capacity (VO_2_ max) and muscle fatty acid β-oxidation [11]. Analysis of the transcript and protein profiles in these patients indicated improvements in oxidative phosphorylation, mitochondrial biogenesis, and angiogenesis, with concomitant reductions in inflammatory and atrophy-related signaling [12]. However, the upstream mechanisms, such as microRNAs, that regulate changes in gene expression in the muscle are unknown.

MicroRNAs, which are small non-coding RNA molecules, are important regulators of gene expression at the post-transcriptional level and function by either inhibiting translation or degrading transcripts once they are bound to a specific seed sequence located in target mRNA. MicroRNAs can target numerous transcripts and have been implicated in the pathogenesis of several rheumatic diseases, including systemic lupus erythematosus and rheumatoid arthritis [13]. In skeletal muscle, microRNAs have been shown to be important during myogenic differentiation [14], and their dysregulated expression has been found in inflammatory myopathies [15]. In particular, pro-inflammatory cytokines have been shown to suppress the expression of microRNAs that are important for muscle differentiation and maintenance, connecting chronic inflammation with muscle degeneration in myositis [16]. The presence of pro-inflammatory cytokines in myositis is partially attributed to activation of the nuclear transcription factor NF-κB [17], which is expressed by both muscle and immune cells and is targeted by glucocorticoids. Therefore, we hypothesized that exercise improves the disease phenotype by altering the expression of microRNAs that target both muscles and immune processes.

## Methods

### Study design

The data collected here are part of a larger randomized, controlled parallel trial evaluating the effect of endurance exercise in myositis skeletal muscle at the Karolinska University Hospital in Stockholm, Sweden titled ‘Physical Exercise as a Targeted Therapy in Patients with Chronic Rheumatic Muscle Disease’ (For full trial protocol, see S1 File) [11, 12] (ClinicalTrials.gov Identifier: NCT01184625). The study followed the World Medical Association’s 2008 Declaration of Helsinki. All participants gave written informed consent to participate in the study, which was approved by the local ethics committee at Karolinksa University Hospital, Sahlgrenska University Hospital and Uppsala University Hospital. The study was approved in August 2006 and February 2009 by the ethics committee. Patient recruitment began in January 2008, the trial was registered with ClinicalTrials.gov in August 2010, and evaluations were performed until August 2012. The authors confirm that all ongoing and related trials for this intervention are registered.

The inclusion criteria included a diagnosis of definite or probable PM or DM for at least 6 months [18, 19], exercising less than once a week, and being on stable medication for at least one month. Patients who were unable to exercise or had severe heart or lung conditions were excluded. Patients were randomized into a control/non-exercise group or an exercise group using a randomization list by an independent nurse [11]. The exercise group performed a 1-hr endurance exercise training program three times a week for 12 weeks as previously described [11]. Muscle biopsies from the vastus lateralis muscle were taken at baseline and after the 12-week training period from both groups under local anesthesia using a semi-open biopsy technique [20]. Tissue (10–80 mg) was taken at different angles in the same incision, immediately frozen in liquid nitrogen-cooled isopentane, and stored at -70°C. De-identified biopsies for molecular analysis were received at Children’s National Medical Center (Washington, DC, USA) under IRB exemption.

Since this study was part of a larger clinical trial examining the benefit of exercise in myositis patients, a total of only three patients were available for analysis. Despite our low sample size, the study was internally controlled, which is important when studying a highly heterogeneous disease with a variable phenotype. Further, although transcriptome and proteome profiles have already been previously published using a larger cohort of patients [12], we repeated both the mRNA microarray using a different platform (i.e. Illumina instead of Affymetrix) and the proteomics using a different approach (i.e. SuperSILAC instead of isobaric mass tagging) in our smaller cohort. These datasets have been uploaded into GEO (Accession Numbers: GSE95735 (microRNA), GSE95772 (mRNA)).

### RNA isolation

Total RNA was isolated from the vastus lateralis muscle at baseline (pre-) and after 12 weeks (post-) of both exercised (n = 3) and control (n = 3) myositis patients using the mirVana^™^ miRNA Isolation Kit (Applied Biosystems/Ambion, Austin, TX, USA) according to the manufacturer’s protocol. The concentration of each RNA sample was determined by a NanoDrop^®^ spectrophotometer ND-1000 (NanoDrop Technologies, Wilmington, DE, USA), and the quality of the RNA samples was assessed with an Agilent 2100 Bioanalyzer (Agilent Technologies Inc., Santa Clara, CA, USA).

### Expression profiling

High-quality total RNA (175 ng) was used for both mRNA and microRNA expression profiling. For mRNA gene expression, samples were analyzed using Illumina^®^ Gene Expression BeadChip Array technology (Illumina, Inc., San Diego, CA, USA). Reverse transcription for the first cDNA strand and synthesis of the second cDNA strand, followed by a single in vitro transcription (IVT) amplification that incorporated biotin-labeled nucleotides, was performed with an Illumina^®^ TotalPrep^™^ -96 RNA Amplification Kit (Ambion, Austin, TX, USA). Biotin-labeled IVT product (cRNA, 750 ng) was hybridized to the HumanHT-12v4_BeadChip (Illumina, Inc., San Diego, CA, USA) for 16 hr, followed by washing, blocking, and streptavidin-Cy3 staining according to the Whole-Genome Gene Expression Direct Hybridization protocol (Illumina, Inc., San Diego, CA, USA). The arrays were scanned using a HiScanSQ System, and the decoded images obtained were analyzed by the GenomeStudio^™^ Gene Expression Module, which is an integrated platform for data visualization and analysis (Illumina, Inc., San Diego, CA, USA). For microRNA expression, samples were analyzed using the Affymetrix GeneChip (Affymetrix, Santa Clara, CA, USA). Total RNA samples, containing low molecular weight RNA, were biotin-labeled with the FlashTag^™^ Biotin HSR RNA Labeling Kit (Genisphere LLC, Hatfield, PA, USA). The quality of the biotin labeling process was confirmed by using an enzyme-linked oligosorbent assay (ELOSA) (Genisphere LLC, Hatfield, PA, USA). Each high-quality biotin-labeled microRNA sample (21.5 μl) was hybridized to an Affymetrix Gene-Chip^®^ miRNA 3.0 Array for 16 hr according to the Affymetrix protocol. The arrays were washed and stained on an Affymetrix Fluidics Station 400 and scanned with a Hewlett Packard G2500A Gene Array Scanner. The Affymetrix^®^ miRNA QC Tool v 1.1.1.0 (Affymetrix, Santa Clara, CA, USA) was used for data summarization and microarray quality control.

### Data analysis

mRNA expression values were generated in GenomeStudio and automatically uploaded (*plug-in*) into Partek software (Partek Incorporated, St. Louis, MO, USA) for statistical analysis and data visualization. Expression values were normalized using Robust Multi-array Average [21], *log*_*2*_-transformed. In addition, Genome Studio Final Report Table was used in Hierarchical Clustering Explorer 3 (HCEv3) for probe-set filtering, power analysis, and chip-based unsupervised clustering [22]. To generate microRNA expression values, Affymetrix GeneChip-derived CEL intensity files were analyzed using the Affymetrix Expression Console Summarization Probe set algorithm for microRNA -RMA+detection above the background (DABG). The gene expression values were filtered based on average signal values, and only microRNAs with average signal values >20% were accepted for further analysis.

### Statistics and microRNA transcript pairing

Illumina GenomeStudio^™^-derived mRNA probe set signal intensity values (RMA normalized and *log*_*2*_-transformed) as well as microRNA probe sets after RMA+DABG and filtering were used in Partek Genomics Suite, version 6.5 (Partek, St. Louis, MO, USA) for determining differently expressed genes, statistics, and data visualization analysis. For the microRNA data set, post-biopsy values were normalized by subtracting the pre-biopsy values. An unequal variance *t*-test was used to identify transcripts and microRNAs of potential interest. Nothing exceeded the false discovery rate (FDR), so expression values with p<0.01 were considered for the further analysis of the microRNA data, while a p<0.05 was the cutoff for the mRNA data set.

To identify microRNA transcript targets, the Ingenuity Pathways Analysis (IPA) (Ingenuity Systems Inc., Redwood City, CA, USA) MicroRNA Target Filter was used to identify transcripts targeted by changed microRNAs. Partek was used to identify probeset IDs associated with the microRNA target genes. Statistics, as described for microRNA identification, were run on the microRNA target genes, and data were filtered to include only mRNA-microRNA interactions that showed expression pairings with changes in opposite directions.

### MicroRNA validation using RT-qPCR

Total RNA (50ng) was reverse-transcribed using the High-Capacity cDNA Reserve Transcription Kit (Thermo Fisher Scientific, Waltham, MA, USA) with the random primer mix substituted with RT primers for hsa-miR-196b (002215) and U47 (001223) (Thermo Fisher Scientific, Waltham, MA, USA). Cycling parameters started with 30 minutes at 16°C, followed by 30 minutes at 42°C, and ended with 5 minutes at 85°C. Approximately 8ng of cDNA was pre-amplified using the Taqman PreAmp Master Mix (Thermo Fisher Scientific, Waltham, MA, USA) following manufacturers protocol with the above primers listed. Amplified cDNA (0.2ng) was loaded in triplicate and run on the 7900HT Fast Real-Time PCR system (Thermo Fisher Scientific, Waltham, MA, USA). Pre Ct values were subtracted from post values for normalization and quantification was performed using the ΔΔCt method (2^-((Normalized Target-Housekeeper)-(Average(Normalized Control-Housekeeper)).

### Proteomics

Total protein was extracted from frozen paired (pre- and post-) biopsies from the exercise group using radioimmunoprecipitation assay buffer (RIPA) buffer (50 mm Tris-HCl, pH 8.0, with 150 mM sodium chloride, 1.0% Igepal CA-630 (Nonidet P-40), 0.5% sodium deoxycholate, and 0.1% sodium dodecyl sulfate) (Teknova, Hollister, CA, USA) containing protease inhibitors (Halt protease inhibitor mixture 100X;Thermo Fisher Scientific, Waltham, MA, USA). Protein concentrations were estimated using the Bio-Rad Microplate Protein Assay (Bio-Rad, Hercules, CA, USA) according to the manufacturer’s protocol.

Approximately 30 μg of each sample RIPA extract was mixed 1:1 with RIPA protein extract from SILAC human myotubes. The SILAC myotubes served as a SuperSILAC internal standard [23], with all lysine residues being replaced with ^13^C_6_,^15^N_2_-lysine and all arginine residues replaced with ^13^C_6_-arginine. Samples underwent detergent removal (Thermo Pierce, Waltham, MA, USA) and tryptic digestion using SmartDigest (Thermo Fisher, Waltham, MA, USA) at 70°C and 1400 rpm for 1.5 hr. The resulting peptides were acidified with 0.1% TFA and fractioned into 8 fractions on high pH reversed-phase spin columns (Thermo Fisher, Waltham, MA, USA). Peptide fractions were dried by vacuum centrifugation and resuspended in 20 μL of 0.1% formic acid and 2% acetonitrile. Each sample (3 μL) was analyzed by top 10 data-dependent LC-MS/MS on a Thermo Q Exactive mass spectrometer coupled online to a NanoEasy Nano-LC 1000 with the following parameters: positive polarity, m/z 400–2000, MS resolution 70,000, AGC 3e6, 100ms IT, MS/MS resolution 17,500, AGC 2e5, 60ms IT, isolation width 3 m/z, and NCE 27, underfill 10%, unassigned and +1 ions excluded, peptide match preferred, isotope exclusion on, dynamic exclusion 25s, LC solvents A: 0.1% formic acid with 2% acetonitrile, B: 100% acetonitrile; precolumn: Thermo Acclaim Pepmap 100 75-μm x 2-cm C18 3-μm 100 A (Thermo Fisher, Waltham, MA, USA); analytical column: Thermo Pepmap RSLC C18 75 μm x 50 cm 100A (Thermo Fisher, Waltham, MA, USA), precolumn equilibration 6 μL at 800 Bar, analytical column equilibration 6 μL at 980 Bar, sample loading 8 μL at 980 Bar; gradient 0%-35% B in 32 min, hold for 2 min, then to 100% B and hold for 10 min. The resulting MS files were searched using the Comet search engine in IP2 software (Integrated Proteomics Pipeline v.3) against the UniProt human database (2014_09, 20,193 reviewed entries) with the following parameters: 2 missed cleavages, partially tryptic, 20-ppm mass accuracy, potential modification of lysine 8.0142 Da, potential modification of arginine 6.0201 Da, and a peptide false-discovery rate of 0.05. Census software version 2014.01 rev. 1, built on the IP2 platform, was used to determine the ratios of unlabeled and labeled peptide pairs using an extracted chromatogram approach. The distribution of ratios was plotted and correction factors applied to adjust for any error in sample mixing. Data were checked for validity by using a regression correlation of better than 0.5 for each peptide pair. A ratio of light/heavy for each protein in each sample was generated and then, using the SuperSILAC approach, the ratio of the pre- and post-samples for each patient was determined. Using this approach, the SILAC standard cancels out to reveal the protein alteration resulting from exercise. Altered proteins were determined using a fold change cut-off of 1.5 and a total peptide count of > = 2.

### Western blot

Total protein lysate (20ug) extracted from pre and post exercised muscle were separated on a 4–12% Bis Tris gel (Thermo Fisher Scientific, Waltham, MA, USA) and transferred for 2 hours at 4°C onto nitrocellulose membranes (Bio-Rad, Hercules, CA, USA). Membranes were blocked for 1 hour using 5% BSA in TBS-0.1% Tween and incubated overnight at 4°C with IκBα (L35A5) mouse monoclonal antibody (1:1000; Cell Signaling Technology, Danvers, MA, USA). Membranes were then washed with TBS-0.1% Tween and probed with anti-mouse IgG, HRP-linked antibody (1:5000; Cell Signaling Technology, Danvers, MA, USA) for 1 hour. ECL chemi-luminescence substrate (Bio-Rad, Hercules, CA, USA) was used to develop blot on ChemiDoc Touch Imaging System (Bio-Rad, Hercules, CA, USA) using the optimal exposure settings. Densitometry analysis was carried out using Image J and ratios of the optical density were normalized to the corresponding loading control (anti-vinculin, 1:1000, Abcam Inc, Cambridge, MA, USA).

## Results

### Study design and patient demographics

To characterize exercise-induced microRNAs in myositis muscle, we made use of samples from PM and DM patients participating in a controlled, randomized trial that examined the benefit of endurance exercise at The Karolinska University Hospital in Stockholm, Sweden ([11], ClinicalTrials.gov Identifier: NCT01184625). These studies have previously shown that endurance exercise benefits PM and DM patients [11, 12]. To better understand the molecular pathways that contribute to exercise-induced benefits, we used muscle biopsies from a subset of patients for the microRNA analysis described here [11].

**Fig 1:**
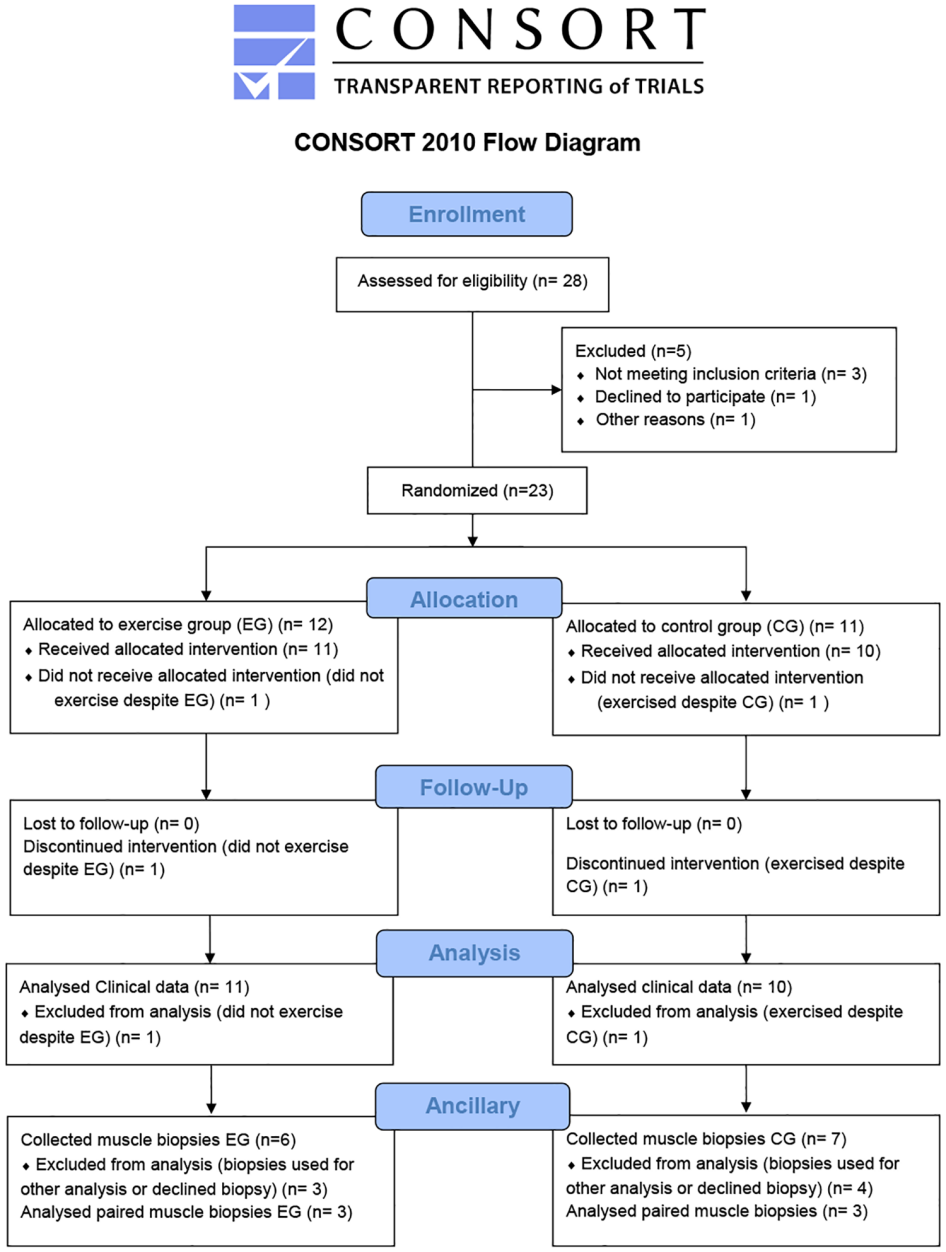
CONSORT 2010 flow diagram. The flow chart depicts the numbers of participants who were randomly assigned, exercised, and were analyzed for study.

**Fig 2:**
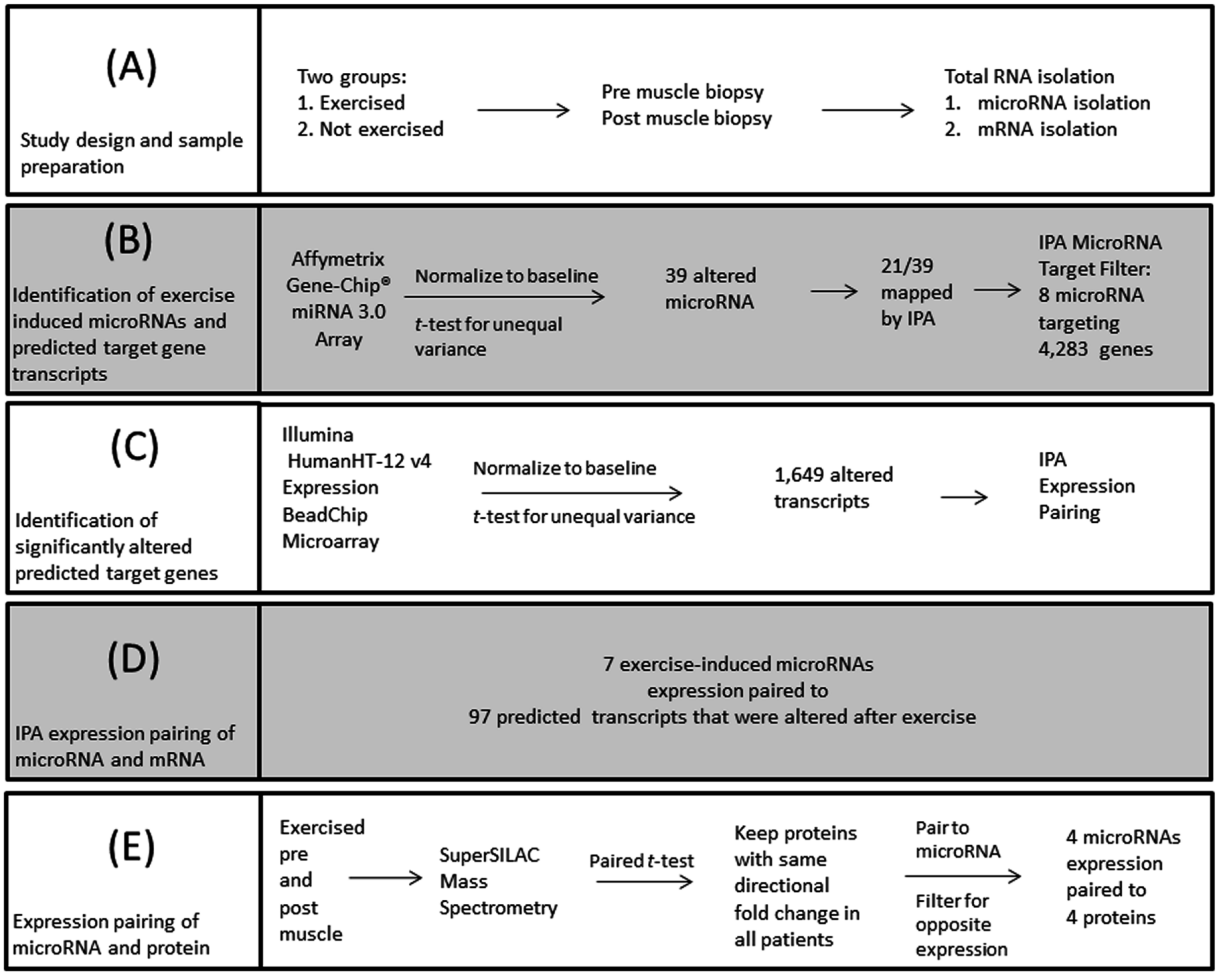
Overview of the data analysis of exercised and non-exercised myositis patients. The above work plan was used to the identify exercised-induced microRNA interactions in myositis patients. (A) Total RNA was extracted from baseline (pre) and exercised (post) muscle biopsies from the exercise group and the control group. (B) After pre values were subtracted from post values as an internal control measure, a total of 39 microRNAs were identified. Ingenutiy Pathway Analysis (IPA) MicroRNA Target Filter identified that 8 of these microRNAs had predicted transcript targets. (C) A gene expression microarray was run to identify if any of these transcript targets were altered in the patients after exercise. (D) Expression pairing between the microRNA and mRNA data sets was performed using IPA to determine biological relevance. (E) Protein was extracted from pre and post exercised muscle for SuperSILAC mass spectrometry. Expression pairing was again performed with microRNA and protein data sets since microRNAs are known to inhibit translation.

Patients were randomized into two groups: an exercised and a non-exercised group (Figs 1 and 2A). All the patients were female, except for one male in the non-exercised group. Each group was composed of one dermatomyositis and two polymyositis patients. The mean age of the exercised group was 63 years, and the average length of disease duration since diagnosis was 1.67 years. The mean age of the control group was 51 years, and the average length of disease duration since diagnosis was 15 years. All patients were on a stable regimen of medication for at least one month prior to the start of the trial. During the trial, all patients were taking prednisone in combination with azathioprine, methotrexate, or mabthera.

### Identification of exercise-induced microRNAs and predicted target gene transcripts

First, the data were internally controlled by subtracting out the baseline or the values obtained from the pre muscle biopsy (i.e. post values–pre values). Statistical analysis was then performed with Partek Genomics Suite, version 6.5 (Partek, St. Louis, MO, USA). To control the false-discovery rate associated with statistical analysis of microarray data, p-values adjusted for multiple corrections were first obtained. This calculation resulted in no significantly altered microRNAs. To obtain a useable dataset, data were filtered to include microRNAs that met two of the following conditions: fold change >|1.2| and p<0.01., Filtering identified 39 microRNAs (S1 Table and Fig 2B). IPA mapped 54% (21/39) of these microRNAs (Table 1), whereas the remaining 36% are currently un-annotated and were therefore excluded from further analysis.

**Table 1:** Exercise-induced microRNAs.

Transcript ID	Fold Change (Exercise (n = 3) vs. Control (n = 3)
hsa-miR-376a-star	-1.6
hsa-mir-3689d-2	1.7
hsa-miR-182	1.3
hsa-mir-630	1.3
hsa-mir-30c-2	1.2
hsa-mir-744	-1.2
hsa-mir-2114	-1.2
hsa-mir-4640	1.3
hsa-miR-2467-5p	1.4
hsa-miR-2278	1.3
hsa-miR-3713	-1.2
hsa-miR-196b	1.6
hsa-mir-3191	1.5
hsa-mir-3654	1.4
hsa-mir-548am	-1.3
hsa-miR-582-5p	-1.3
hsa-mir-3166	1.5
hsa-mir-133b	-1.4
hsa-miR-548d-5p	-1.5
hsa-mir-609	1.4
hsa-mir-4295	1.5

To identify transcript targets, we used IPA’s MicroRNA Target Filter because it pulls predicted gene targets from multiple databases and allows for prioritization of experimentally validated interactions. IPA’s MicroRNA Target Filter predicted 4,283 gene targets from 32% (8/25) of the mapped microRNAs (S2 Table).

### Identification of altered predicted target gene transcripts after exercise

To identify if any of the predicted 4,283 gene targets from the mapped microRNAs were altered after exercise in our cohort of patients, an Illumina gene expression microarray was run and altered transcripts were determined using the same analysis for identification of exercise-induced microRNAs with the exception of the cut off for the p value (Fig 2C). To determine biologically meaningful interactions, microRNA-mRNA pairs were filtered based on expression pairing (Fig 2D). This analysis identified 7 exercise-induced microRNAs with 97 predicted transcript targets displaying a fold change in the opposite direction from that of their matched microRNAs (Table 2). Further, pulling from IPA’s compiled literature database, thirty-two of these transcript targets had previously been documented to participate in over 400 various signaling pathways (S3 Table).

**Table 2:** Overview of microRNA expression pairing with mRNA and proteomic data sets.

Transcript ID	Seed Sequence	Fold Change (Exercise (n = 3) vs. Control (n = 3)	Total predicted transcript targets	Transcripts altered due to transcript degradation	Proteins altered due to translational repression
hsa-miR-376a-star	UAGAUUC	-1.65	176	1	1
hsa-miR-182	UUGGCAA	1.3	1164	44	0
hsa-miR-2467-5p	GAGGCUC	1.38	840	17	0
hsa-miR-2278	AGAGCAG	1.31	374	12	0
hsa-miR-196b	AGGUAGU	1.59	611	17	0
hsa-miR-582-5p	UACAGUU	-1.32	558	4	1
hsa-miR-548d-5p	AAAGUAA	-1.52	429	2	3
hsa-miR-3713	GUAUCCG	-1.2	131	0	1

To determine biological function, we used IPA’s canonical pathway analysis and found that microRNA-mRNA expression pairs were involved in pathways affecting immune response, capillary growth, muscle metabolism, and muscle atrophy (Table 3).

**Table 3:** Pathway analysis of expression-paired microRNA-mRNA alterations in exercised patients.

ID	Fold Change (Exercise (n = 3) vs Control (n = 3)	ID	Fold Change (Exercise (n = 3) vs Control (n = 3)	IPA Identified Pathway	Function
hsa-miR-182	1.304	*BCL2*	-1.5	PEDF Signaling	Anti-angiogenic, Immune response
		*BDNF*	-1.5		
		*GDNF*	-1.2		
		*PIK3R1*	-1.3		
hsa-miR-196b	1.595	*IKBKB*	-1.5		
		*GDNF*	-1.2		
hsa-miR-2467-5p	1.379	*GDNF*	-1.2		
hsa-miR-182	1.304	*CREB1*	-2.3	PI3K Signaling in B Lymphocytes	Immune response
		*ATF7*	-1.2		
		*PIK3R1*	-1.3		
hsa-miR-196b	1.595	*IKBKB*	-1.5		
hsa-miR-182	1.304	*TNFSF14*	-1.7	Lymphotoxin β Receptor Signaling	Immune response
		*PIK3R1*	-1.3		
hsa-miR-196b	1.595	*IKBKB*	-1.5		
hsa-miR-182	1.304	*KDELR1*	-1.7	Protein Kinase A Signaling	Glucose, Protein, and Lipid Metabolism
		*PDE7A*	-1.8		
		*CREB1*	-2.3		
		*ADD3*	-1.1		
hsa-miR-2467-5p	1.379	*PPP1R1B*	-1.6		
hsa-miR-182	1.304	*CREB1*	-2.3	Glucocorticoid Receptor Signaling	Muscle atrophy, Immune response
		*BCL2*	-1.5		
		*PIK3R1*	-1.3		
hsa-miR-196b	1.595	*IKBKB*	-1.5		
hsa-miR-2278	1.308	*SUMO1*	-1.5		

### Identification of altered predicted target genes at the protein level after exercise

To determine how exercise-induced microRNAs affect expression at the protein level, global mass spectrometry using the SuperSILAC approach was performed on muscle from the exercised patients (Fig 2E) [23]. Altered proteins were filtered to only include those with a fold change in the same direction in all three patients after exercise (S4 Table). This analysis resulted in 44 proteins that were changed after endurance exercise. These proteins were then compared to IPA’s MicroRNA Target Filter Function (Fig 2B) to determine if any of the proteins were predicted to be microRNA targets. Expression pairing revealed that 9% (4/44) of the altered proteins were predicted microRNA targets (Table 2). To determine biological function, we again pulled from IPA’s compiled literature database for pathway identification. Altered proteins that were associated with changes in their respective target microRNAs were involved in pathways affecting mitochondrial biogenesis, oxidative stress, and muscle remodeling (Table 4).

**Table 4:** Pathway analysis of expression-paired microRNA-protein alterations in exercised patients.

ID	Fold Change (Exercise (n = 3) vs Control (n = 3)	Symbol	Fold Change (Exercise (n = 3) vs Control (n = 3)	IPA Identified Pathway	Function
hsa-miR-3713	-1.19	AK3	1.86	AMPK Signaling	Mitochondrial biogenesis [24]
hsa-miR-548d-5p	-1.52				
hsa-miR-548d-5p	-1.52	APEX1	2.697	BER pathway, cAMP-mediated, β-adrenergic, HIF1α, Protein Kinase A, and Relaxin Signaling	Protects against oxidative stress [25]
hsa-miR-548d-5p	-1.52	HIBADH	1.972	Valine Degradation I	Mitochondrial biogenesis [24]
hsa-miR-376a-star	-1.65	CAP2	1.758		Actin binding, Muscle remodeling [25]
hsa-miR-582-5p	-1.32				

### Increased expression of miR-196b after exercise is associated with increases in total IκBα protein

To further explore how microRNA may mediate disease improvements, we chose to focus on the microRNA-mRNA pair, miR-196b and *IKBKB* for several reasons. First, NF-κB activation, which is a known regulator of skeletal muscle inflammation in myositis, occurs downstream of *IKBKB*, which transcribes the beta subunit (IKK-β) for the IKK enzyme complex. Second, *IKBKB* is essential for classical NF-κB signaling, which induces proinflammatory cytokine production. Third, when filtering for experimentally validated interactions using the IPA filter (i.e. previously published studies showing regulation using a luciferase assay, RT-qPCR), miR-196b and *IKBKB* remain. Our expression-pairing analysis concluded that increases in miR-196b were associated with decreased expression of known transcript target *IKBKB* in patients after exercise.

**Fig 3:**
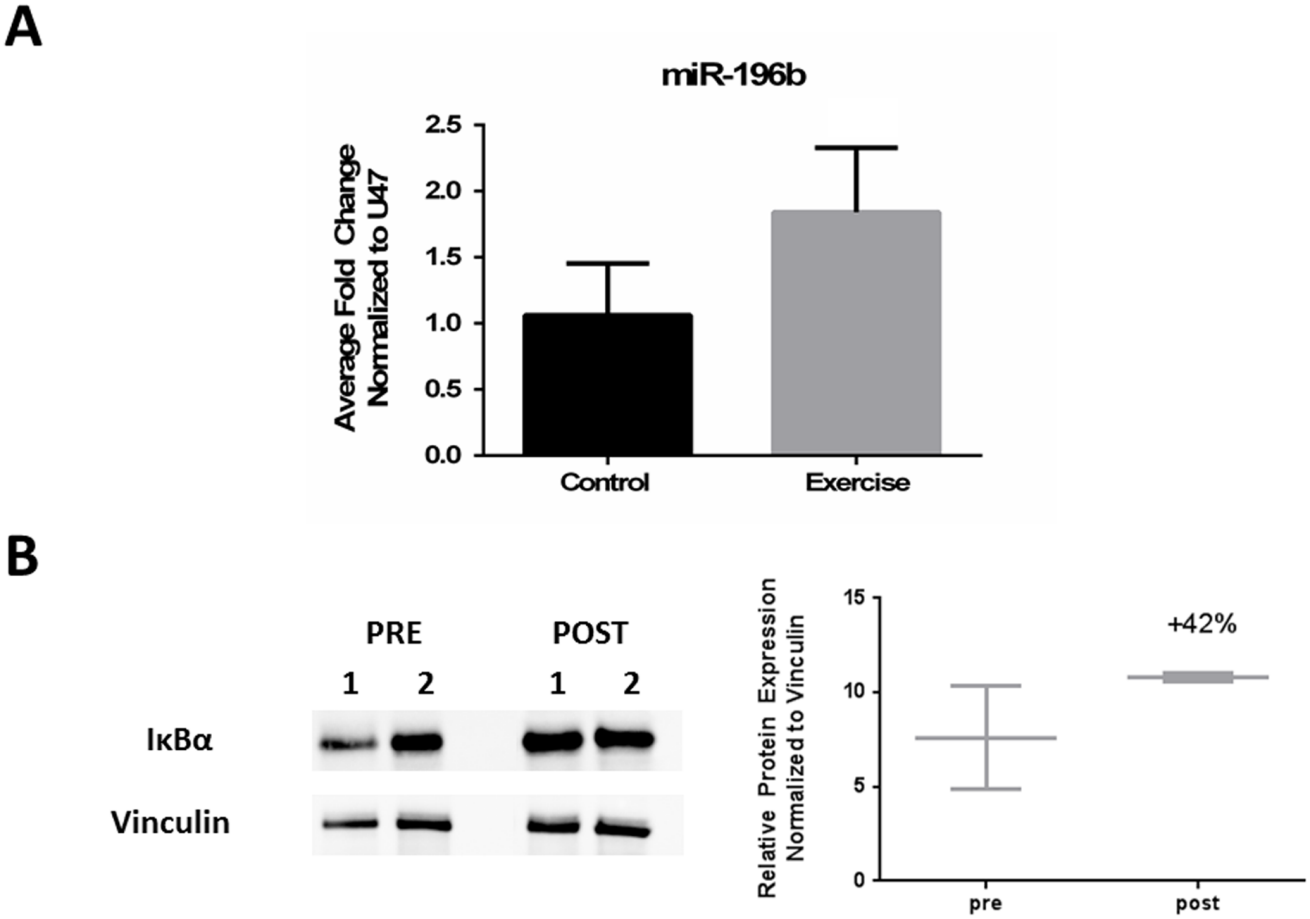
Validation of miR-196b expression after exercise and its associated effect on classical NF-κB signaling. (A) RT-qPCR validated that miR-196b was increased after exercise. For normalization, pre Ct values were first subtracted from post Ct values from both miR-196b and the housekeeper, U47. Double delta Ct method was then used to calculate the average fold change (2^-((Normalized Target-Housekeeper)-(Average(Normalized Control-Housekeeper)). (B) Western blot in pre-and post exercised skeletal muscle shows an average increase of 42% in total IκBα protein after exercise.

We first validated that miR-196b was increased after exercise by using another method, RT-qPCR, to measure microRNA expression (Fig 3A). To determine if increased miR-196b expression was associated with decreases in classical NF-κB signaling, we immunoblotted for total IκBα protein. IκBα functions as an inhibitor of NF-κB by keeping NF-κB in its inactive state through sequestration within the cytoplasm and blocking its ability to bind to DNA. Further, IκBα protein levels are regulated through degradation induced by IKK-β (*IKBKB*). We found a variable response in total IκBα protein between patients before and after exercise. Averaged total protein levels showed a trend towards increasing after exercise (Fig 3C), suggesting less degradation of IκBα and attenuated classical NF-κB signaling.

## Discussion

Exercise has been shown to enhance skeletal muscle function by increasing strength and contractility and reducing negative effects (i.e., oxidative stress, inflammation) associated with muscle disease [7]. Since exercise directly targets the muscle, myositis patients were traditionally advised not to exercise out of fear for exacerbating existing muscle damage and inflammation. However, substantial evidence has shown that a consistent exercise regimen exerts anti-inflammatory effects and may be beneficial to individuals with multiple chronic inflammatory conditions [6]. In myositis, exercise provides clinical benefit and is an attractive therapy because it non-invasively targets skeletal muscle with relatively few to no side effects [8, 9, 11, 12]. Previous studies have tried to link clinical improvements with underlying molecular mechanisms; however, these studies have only examined global gene and protein changes [12]. Currently, there are no studies that examine how exercise affects potential genetic regulators, such as microRNAs. This is the first study that has tried to integrate exercise-induced microRNA alterations with subsequent target gene and protein expression.

**Fig 4:**
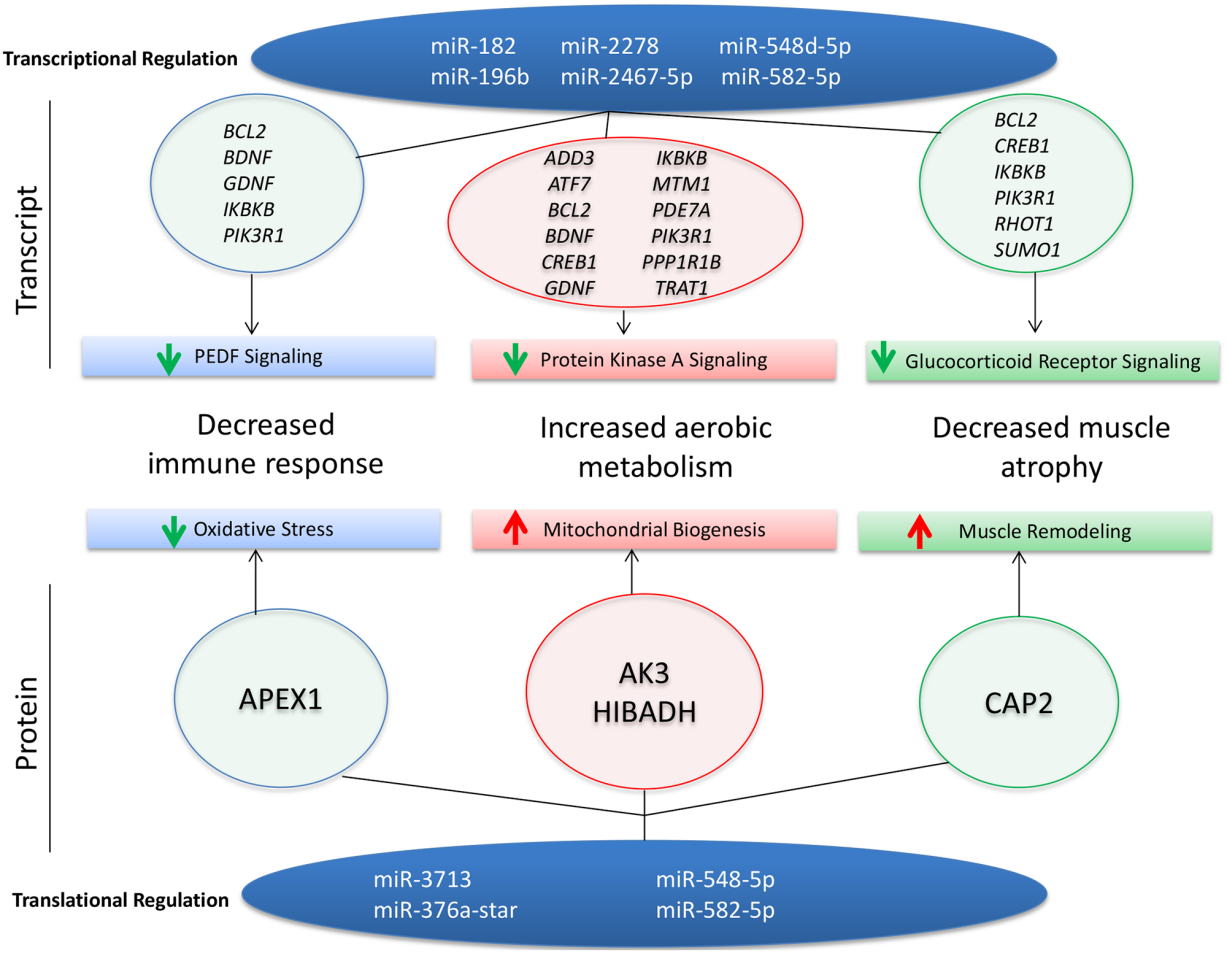
Exercise induces microRNAs that target transcripts and proteins important for muscle and immune response. Exercise-induced microRNAs help improve disease outcomes in myositis by modulating transcripts and proteins important for aerobic metabolism, immune response, and muscle atrophy.

One of the major issues in studying rare neuromuscular diseases is the heterogeneity of the patient population. A main strength of this study is the experimental setup in which skeletal muscle was taken from paired muscle biopsies of myositis patients before and after an aerobic exercise intervention. This controlled for the wide variability seen in the disease phenotype since each patient was normalized to its baseline level. Additionally, there are two previously published studies using larger, separate cohorts of endurance exercised patients so we were able to use an evidence-based approach by cross-referencing both array and functional data [26]. Since our cohorts were different, we repeated both transcriptomics using a different platform (Illumina instead of Affymetrix) and proteomics using a different approach (SuperSILAC instead of isobaric mass tagging). We hypothesized that microRNAs regulate processes involved in the both the muscles and the immune system. Specifically, we found that exercise induced the expression of microRNAs that targeted mRNA transcripts as well as proteins involved in suppressing inflammation, promoting muscle growth, and improving aerobic respiration in skeletal muscle after a 12-week endurance-exercise intervention in myositis patients (Fig 4). Our findings suggest that exercise-induced microRNAs help to regulate the genetic, proteomic, and functional benefits previously reported [11, 12].

Pathway analysis of exercise-induced changes in microRNA revealed that exercise-induced microRNAs primarily down-regulate transcripts involved in immune response, glycolytic metabolism, and muscle atrophy. This suggests that skeletal muscle inflammation is partially regulated by microRNAs in myositis. To further explore this, we used targeted gene expression analysis and found that *IKBKB* mRNA was reduced following exercise training, in accordance with increased mir-196b expression (Table 3). *IKBKB* is an upstream modulator of NF-κB and assists in its translocation to the nucleus by marking NF-κB inhibitors (IκBα) for degradation by proteosomes. When compared to normal muscle, myositis muscle shows activated NF-κB translocation to the nucleus, as well as increases in NF-κB target genes [17]. Interestingly, the majority of the identified transcriptional pathways have been shown to regulate NF-κB signaling [27, 28]; therefore, we focused on further study of the microRNA-mRNA pair, miR-196b and *IKBKB*.

Although we confirmed that miR-196b was increased in the exercised group by using RT-qPCR, the interpretation of how this affects NF-κB signaling within our cohort of patients remains less clear due to the limitations of sample availability, small sample size, and an absence of healthy control subjects. Further, there was a variable response between patients when examining total amounts of IκBα protein after exercise. To further explore the effect of exercise on this pathway, a larger cohort of myositis patients is needed.

We further explored how microRNAs influence the regulation of protein expression, since microRNAs are known to influence gene expression through either transcriptional degradation or translational repression. We established that most the proteins regulated by microRNAs (AK3, HIBADH) are localized within mitochondria and are suggestive of increased mitochondrial biogenesis. This is in agreement with previous studies that have shown increases in the aerobic respiration of exercised patients [11, 12]. Furthermore, the microRNAs that were predicted to target these proteins were downregulated (Table 3), suggesting that microRNAs regulate aerobic metabolism and could contribute to patients’ functional improvements in aerobic respiration after endurance exercise [11]. The number of proteins altered by exercise involved in inflammatory processes (Table 4 and Fig 4) was much lower than at the transcript level. This is most likely due to limitations within our method for protein detection. Many of the inflammatory proteins were not present as we used healthy myotubes as an internal standard. Despite this, we were able to detect a possible microRNA-mediated increase in APEX1, which has been previously shown to relieve oxidative stress during an acute bout of exercise in skeletal muscle [25].

In summary, exercise induces changes at the microRNA level that target genes at both the transcriptional and protein levels that are implicated in immune responses, aerobic metabolism, and muscle atrophy (Fig 4). Interestingly, similar effects have been reported in healthy individuals after exercise [7, 12, 21, 29], suggesting that myositis muscle may adapt to training in a manner that is comparable to healthy muscle. This could suggest that inflammation is a driving factor in disease pathogenesis; however, several studies have shown that inflammation is poorly correlated with myositis muscle pathology [30–32], which indicates a role for non-immune mechanisms in disease pathogenesis. Therefore, it is possible that exercise targets not only inflammatory pathways, but also additional non-immune pathogenic pathways implicated in the muscle. This is supported by studies showing that myositis patients on immunosuppressive therapies have impairments in aerobic capacity when compared to healthy age and physical activity matched controls [11]. In this case, although exercise has similar effects in both healthy and myositis muscle, exercise becomes therapeutic because it targets both immune and non-immune mechanisms implicated in disease. However, further studies comparing exercise in healthy control and myositis muscle are needed to answer this question.

Because of the side effects of glucocorticoids, patients can only take small dosages that aim to balance the treatment of symptoms with negative off-target effects associated with prolonged use. Because of these dosage restrictions, the dosage may be sufficient to clear systemic inflammation that causes the infiltration of immune cells into the muscle, but it may not be high enough to target NF-κB activation in the muscle effectively without producing severe side effects such as muscle atrophy and wasting. Therefore, exercise may be able to target NF-κB in the muscle better than the current doses of glucocorticoids can, and this improved targeting could help to explain the overall improvement in patients after endurance exercise. Endurance exercise may act as a glucocorticoid-sparing intervention, or it may supplement and work in combination with glucocorticoid usage to permit patients to receive lower doses, reduce unwanted side effects, and give maximal benefit for disease treatment. Further studies validating these microRNAs in a separate larger cohort of exercise patients are needed to determine significance and to further understand the molecular mechanisms that result in disease improvement after exercise.

## Supporting information

S1 Table Altered microRNAs after endurance exercise in myositis patients. This table shows the full data set of microRNAs that were found to be altered after 12 weeks.
(CSV)

S2 Table IPA’s MicroRNA target filter results. This table shows the full list of transcripts that the mapped microRNAs are predicted to target.
(CSV)

S3 Table Expression pairing of microRNA with mRNA in exercised patients. This table associates the changes in transcript expression to changes in microRNA expression.
(CSV)

S4 Table Altered proteins after endurance exercise. This table shows the full data set of protein changes that were identified by mass spectrometry.
(CSV)

S1 File Trial study protocol. (PDF)

S2 File CONSORT 2010 checklist. (DOC)
