# Supplementary material for: Effect of endurance exercise on microRNAs in myositis skeletal muscle—A randomized controlled study
Source: PLoS One. 2017 Aug 22;12(8):e0183292. doi: 10.1371/journal.pone.0183292 (PMC5568726; doi:10.1371/journal.pone.0183292)
Supplement: S1 File — (PDF) [file pone.0183292.s005.pdf]

Bilaga 2

Exercise as a targeted therapy in patients with inflammatory myopathies, myositis – effects on disability and muscle characteristics.

The over all aim of this study is to use exercise as a targeted therapy to achieve improved understanding of disease mechanisms in inflammatory myopathies (IIM), myositis and to evaluate effects of a muscular endurance training in muscle tissue as well as on disability.

SPECIFIC GOALS

The specific objectives of this study are to test the hypothesis that hypoxia is an important factor contributing to clinical symptoms. This hypothesis will be addressed by two approaches. The first will be to evaluate surrogate markers for hypoxia, such as lactate, and muscle characteristics in skeletal muscle during rest and after an endurance exercise bout in patients with chronic polymyositis (PM) / dermatomyositis (DM). Based on the assumption that endurance training will improve hypoxia, the second approach will be to evaluate effects of muscular endurance training on metabolites and molecular expression in skeletal muscle tissue as well as disability following a 12-week endurance training program for patients with chronic PM/DM.

BACKGROUND

Polymyositis (PM) and dermatomyositis (DM) are included in the idiopathic inflammatory myopathies which are rare chronic rheumatic muscle disorders characterized by slowly progressing impairment of muscle endurance and muscle weakness together with fatigue. If untreated, a majority of the myositis patients become wheel-chair bound and may need assisted ventilation. About 30-60 % of the patients develop lung fibrosis. (Henriksson *and* Lindvall 1990). Diagnosis is set according to established criteria; Specific inflammatory infiltrates in muscle tissue, muscle weakness, increased phosphocreatine kinase levels in serum (CK), characteristic EMG-changes and skin rashes in DM (Bohan & Peter 1975). Medical treatment of today in patients with PM and DM consists of high-dose corticosteroids together with immunosuppressive agents. Despite positive effects of treatment most patients develop sustained disability (Marie I *et al* 2001). Patients with PM and DM have significantly poorer perceived health compared to healthy individuals (Sultan *et al* 2002) and patients with rheumatoid arthritis regarding domains Physical, Social and Energy (Chung *et al* 2001).

The mechanisms leading to muscle impairment are not yet fully understood. Patients with PM and DM have decreased number of capillaries in muscle tissue and activation of endothelial cells and increased expression of cytokines such as (IL)-1 and transforming growth factor (TGF)-beta in muscle tissue (Englund *et al* 2002). All these changes could be induced by hypoxia. An increased expression of vascular endothelial growth factor (VEGF), also indicating a state of hypoxia in muscle in patients with PM/DM has recently been reported from our research group (Grundtman *et al* 2004).

Moreover, we have recently demonstrated a reduced percentage of oxidative type I fibers in patients with chronic PM and DM also indicating muscle tissue hypoxia. Interestingly, an increased number of type I fibers along with improved muscle function was achieved after a sub-maximal 12-week home exercise program suggesting improvement of microcirculation as a result of exercise (Dastmalchi *et al* unpublished data). There are no methods to assess hypoxia directly in muscle tissue. However, a microdialysis technique using multiple microelectrodes is available at the Karolinska Institutet, Stockholm, Sweden which can be used to detect local metabolic effects of insulin and insulin permeability (Gudbjörnsdottir et al 2005) as well as lactate concentrations in skeletal muscle (Lundberg *et al* 2001). However, no studies with the objective to evaluate oxygen tension in skeletal muscle tissue during and after exercise in patients with PM or DM have been carried out.

For many years, patients with inflammatory myopathies were discouraged from physical activity and exercise due to fear of increased muscle inflammation. However, during the last decade a few small studies evaluating safety and efficacy of different exercise programs have reported no signs of increased muscle inflammation but instead decreased disability, however, so far not reaching normal function. Our research group reported positive effects on muscle impairment and perceived health in patients with chronic, stable as well as in patients with recent onset, active PM and DM (Alexanderson *et al* 1999, 2000). A 60-minute aerobic exercise program on an intensity of 60% of maximal heart rate produced increased aerobic capacity, muscle strength and decreased activity limitation in patients with chronic, stable PM/DM (Wiesinger *et al* 1998a,b). In collaboration with King's College Hospital, London we reported positive effects on muscle impairment by a home exercise program in combination with creatine supplements compared to exercise alone in chronic PM/DM (Alexanderson *et al* 2004).

It is crucial to improve knowledge of mechanisms causing the sustained muscle weakness to improve the care of patients with myositis. Most previous studies evaluating different exercise regimens in these patients are open studies including a limited number of patients. However, they indicate beneficial effects of short-term exercise on disability and molecular parameters. Further randomized controlled trials including a larger number of patients are needed to increase our understanding of clinical effects of exercise.

METHODS

General proceduresTo address the first objective muscle metabolites in skeletal muscle tissue of patients with PM and DM will be compared to matched healthy controls with lactate/pyruvate as primary outcomes. The effects of an endurance exercise program on muscle metabolites

and function will then be evaluated in a randomized controlled trial with lactate/pyruvate and muscle endurance as primary outcomes.

Assessments

Muscle metabolites, muscle characteristics and disease activity

Lactate / pyruvate, glycerol and muscle metabolites such as adenosine triphosphate / adenosine diphosphate (ATP/ADP) will be measured using a microdialysis technique. Two thin catheters with a 2 cm long membrane with a diameter of 0.5 mm (CMA 63) will be inserted in the vastus lateralis 20 cm proximal to the knee joint, 2 cm apart, under local anesthesia (Lundberg et al 2001). A reference catheter will also be inserted subcutaneously in the belly. A probe is perfused with a physiological solution with 2 μl/min. Water soluble substance in the interstitial fluid will diffuse across the semipermeable dialysis membrane and enter the perfusate. After 90 minutes equilibration time, when 2 perfusates/catheter are collected, a 30-minute cycle bout will be performed on a load of 30% of maximal healt rate for patients and 70% of maximal heart rate for healthy controls. During this time 3 perfusates are collected. The patient will then rest for another 60 minutes while the last 2 perfusates are collected. The perfusates will then be frozen in -20°. Lactate, pyruvate and glycerol will later be analyzed using a Clinical Microdialysis Analyzer (CMA, Solna, Sweden). ATP/ADP will be analyzed at the rheumatology lab, Karolinska University Hospital.

Muscle biopsies of the vastus lateralis will be taken under local anesthesia using a chonchotome in patients who accept a muscle biopsy. Three to 6 specimens of 10-80 mg each will be taken from different angles in the muscle tissue in the same incision. The second biopsy will be taken from the contra lateral side. Analysis include histopathology, biochemistry and immunohistochemistry for inflammatory cells, cytokines and other inflammatory molecules, ATPase stainings for muscle friber composition, cross sectional area and analysis of number of capillaries and growth hormones. These analyses will be performed at the rheumatology research lab at the Centrum for Molecular Medicine, Department of Neuropathology and at the Department of Clinical Physiology at Karolinska University Hospital (Dorph *et al* 2001). The muscle biopsies will also be analyzed with a micro-array technique and with a new proteomics technique. The micro-array analysis will be performed at the Children's hospital in Washington DC in collaboration with Professor K Nagaraju, thereafter confirmatory studies on protein expression will be performed at the Karolinska University Hospital.

A validated core set of measures to assess disease activity; physicians / patients assessment of global disease activity on a visual analogue scale (VAS), muscle strength by manual muscle test (MMT), Physical function by the Health Assessment Questionnaire (HAQ), laboratory assessments of muscle enzymes and assessment of extra-skeletal involvement (Miller *et al* 2001).

Bilaga 2

Blood samples (20 ml/occasion) will be taken for analysis of muscle enzymes, inflammatory markers, cytokines.

Impairment

A submaximal oxygen uptake test will be performed on a stationary bike, the Åstrand 6-minute test. Before starting the test, the patients' age, weight and gender are registrated in a computer and the patient is connected to an electronic heart rate measuring device. The tests starts at 300 kpm for women and 450 kpm for men. Intensity os increased during the first two minutes until the patients heart rate exceeds 120 bpm. The the patient continues to work on the same intensity for antoher four minutes. Central exertion is rated on the Borg RPE, 6-20, scale every second minute. After completed test the estimated oxygen uptake in ml/kg/min is registrated.

Functional Index-2 (FI-2) is a valid and reliable disease specific index measuring muscle endurance in patients with PM/DM. The FI-2 contains seven functional tasks each scored as the number of correct performed repetitions, from 0 = severe impairment to 60/120 = no impairment (Alexanderson *et al* 2006).

Five voluntary repetition maximum (VRM) of knee extensors sitting with full thigh support testing from 90° knee flexion up to 0° flexion will be used to measure dynamic muscle strength of the quadriceps.

Activity / participation

The Myositis Activities Profile (MAP) is a valid and reliable disease specific questionnaire measuring activity limitation for patients with PM and DM. The MAP consists of 31 activities divided in to four subscales; Movement, Activities of moving around, Self-care, and Domestic and four single items; Social, Avoiding over exertion, Work, and Leisure. Each activity is scored on a seven grade scale from 1 = no troubles at all to 7 = impossible to do. Total score of each subscale is the median value of all scores within each subscale (Alexanderson *et al* 2002).

The SF-36 is a generic self-administrated questionnaire measuring perceived health consisting of eight sub-scales; Physical functioning, Role physical, Bodily pain, General health, Vitality, Social functioning, Role emotional, and Mental health. Each sub-scale is scored from 0-100, where 100 indicates good health (Sullivan *et al* 1995).

Bilaga 2

Patients and healthy controls

Patients

All patients with chronic PM/DM registered at the Rheumatology clinic, Karolinska University Hospital, Stockholm, meeting the inclusion criteria will be invited to participate.
Inclusion criteria: a) diagnosis PM/DM according to Bohan and Peter criteria (Bohan and Peter 1975), b) age 18-80 years, c) diagnosis duration > 6 months d) exercising ≤ once a week, e) unchanged disease activity and medication since one month. Exclusion criteria: a) severe lung fibrosis, b) heart or lung condition contraindicating vigorous exercise, c) severe osteoporosis, d) anti-coagulant therapy.

Healthy controls

For each patient included a healthy age and gender matched healthy control will be identified.
Inclusion criteria: a) age 18-80 years, b) exercising ≤ once a week. Exclusion criteria: a) Chronic musculoskeletal disorder, b) severe osteoarthritis or osteoporosis, c) heart or lung condition contraindicating vigorous exercise, d) history of bleeding tendency.

Experimental procedures

Four weeks prior to training clinical assessments of disease activity, blood samples and also muscle biopsies will be performed. One week later the microdialysis investigations will take place. The following week a learning occasion of the functional measures of muscle strength and endurance as well as a submaximal oxygen uptake test will be performed. One week later tests of muscle function and oxygen uptake will be performed. Thereafter, the patients will be rancomized to either an exercise group (EG) or a control group (CG). The EG will perform a one-hour exercise program containing a 5-minute warm-up on 50% of estimated maximal heart rate followed by 30 minutes cycling on 70% of maximal heart rate and 20 minutes of muscular endurance training on both upper and lower limbs at about 50% of 1 voluntary repetition maximum (VRM). The program ends with cool-down and stretch. During the first two weeks of the study training intensity will be gradually increased from 50% up to 70% of the patients' individual maximal heart rate. The EG is planned to exercise altogether three times a week during 12 weeks, twice a week at the Physical Therapy Department and once a week with a similar program at home. The CG will not participate in any intervention but will later on be invited to perform the vigorous endurance-training program. All training sessions at the clinic will be supervised by the same physical therapist and all patients will also receive an exercise diary to register their home exercise and physical activity during the exercise periods and the follow-up months.

Independent physical therapists and physicians will perform all assessments. Follow-up microdialysis investigation and muscle biopsies before and after an exercise bout will be performed after 12 weeks of exercise. Blood samples and assessments of disease activity and disability will be performed at baseline and after 12, 24, 36 and 52 weeks. Assessments of muscle muscle strength and

endurance will be performed by an independent physical therapist. Questionnaires (MAP and SF-36) will be administered by an independent nurse.

Data on disability will be analyzed on group level as well as by international criteria for minimal clinical improvement set for patients with IIM: To be a responder to treatment a patient should improve ≥ 20% in 50% of measures used with no more than 2 worse by ≥ 25% (which could not include measure of muscle impairment). A power analysis indicates that at least 15 patients in each group as well as 10 healthy controls are acquired to reach statistical power.

Significance

The use of the microdialysis for analysis of surrogate markers for hypoxia and muscle metabolites in muscle in relation to exercise might lead to improved understanding of disease mechanisms and therefore lead to improved therapies for patients with PM and DM. The exercise study could is likely to contribute to improved knowledge of effects of endurance training.

References

- Alexanderson H, Stenström CH, Lundberg I. Safety of a home exercise programme in patients with polymyositis and dermatomyositis. Rheumatology 1999;38:608-11.
- Alexanderson H, Stenström CH, Lundberg IE. The safety of a resistive home exercise program in patients with recent onset active polymyositis or dermatomyositis. Scand J Rheumatol 2000;29:295-301.
- Alexanderson H, Lundberg IE, Stenström CH. Development of the Myositis Activities Profile – validity and reliability of a self-administrated questionnaire to assess activity limitations in patienter with polymyositis/dermatomyositis. J Rheumatol 2002;29:2386-92.
- Alexanderson H, Broman L, Tollbäck A et al. The functional index-2, FI-2, a valid and reliable measure of impairment in patients with polymyositis and dermatomyositis. Submitted 2004.
- Alexanderson H, Chung YL, Pipitone N et al. Creatine Supplements Improve Muscle function in idiopathic inflammatory myopathies in a 6-month double-blind, randimised placebo-controlled trial. Arthritis Rheum 2004;S667:1785.
- Bohan A, Peter JB. Polymyositis and dermatomyositis (first of two parts). N Engl J Med 1975;292:344-7.
- Chung YL, Mitchell HL, Houssien DA et al. A comparative study of outcome in myositis and other musculoskeletal disorders assessed using the Nottingham Health Profile. Clin Exp Rheumatol 2001;19:447-50.
- Dorph C, Nennesmo I, Lundberg I. Percutaneous Conchotome muscle biopsy. A useful diagnostic and assessment tool. J Rheumatol 2001;28:1591-9.
- Englund P, Nennesmo I, Klareskog L *et al*. Interleukin 1-alpha expression in capillaries and major histocompatability complex class I expression in type II muscle fibers from polymyositis and dermatomyositis patients: important phatogenic features independent of imfallatory cell clusters in muscle tissue. Arthritis Rheum 2002;46:1044-55.
- Fries JF, Spitz P, Kraines RG et al. Measurement of patients outcome in arthritis. Arthritis Rheum 1980;23:137-45.
- Griggs RC, Askanas V, DiMauri S et al. Inclusion body myositis and myopathies. Ann Neurol 1995;38:705-13.

- Grundtman C, Ulfgren AK, Borg K et al. Dowd-regulation of the abberant expression of the inflammatory mediator high mobility group box chromosomal protein 1 in muscle tissue of patients with polymyositis and dermatomyositis. Arthritis Rheum 2004, accepted.
- Gudbjörnsdottir S, Sjöstrand M, Strindberg L et al. Decreased muscle capillary permeability surface area in type 2 diabetic subjects. J Clin Endocrinol Metab. 2005;90:1078-82.
- Hamrin K, Rosdahl H, Ungerstedt U *et al.* Microdialysis in human skeletal muscle: effects of adding colloid to the perfuse. J Appl Physiol 2002;92:385-93.
- Henriksson KG, Lindvall B. Polymyositis and dermatomyositis 1990 – diagnosis, treatment and prognosis. Prog Neurobiol 1990;35:181-93.
- Lundberg G, Olofsson P, Ungerstedt U et al. Lactate concentrations in human skeletal muscle biopsy, microdialysate and venous blood during dynamic exercise under blood flow restriction. Pflugers Arch. 2002;443:458-65.
- Marie I, Hachulla E, Hatron PY et al. Polymyositis and dermatomyositis: shortterm and longterm outcome, and predictive factors of prognosis. J Rheumatol 2001;28:2230-7.
- Miller FW, Rider LG, Chung YL etal. Proposed preliminary core set measures for disease outcome assessment in adult and juvenile idiopathic inflammatory myopathies. Rheumatology 2001;40:1262-73.
- Sullivan M, Karlsson J, Ware JE. The Swedish SF-36 health survey. Evaluation of data quality, scaling assumptions, reliability and construct validity across general population in Sweden. Soc Sci Med 1995;41:1349-58.
- Wiesinger GF, Quittan M, Aringer M et al. Improvement of physical fitness and muscle strength in polymyositis/dermatomyositis patients by a training programme. Br J Rheumatol 1998;37:196-200.
- Wiesinger GF, Quittan M, Graninger M etal. Benefit of 6 months long-term physical training in polymyositis/dermatomyositis. Br J Rheumatol 1998;37:1338-42.

EPN
REGIONALA ETIKPRÖVNINGSNÄMNDEN · STOCKHOLM ·

Avdelning 1

PROTOKOLL 2006/1:7
2006-08-23

Sammanträde i Stockholm

NÄRVARANDE

Ledamöter
Olof Forssberg, ordförande
Pierre Lafolie, vetenskaplig sekreterare (*klinisk farmakologi*)
Börje Bjelke (*geriatrik*), deltar inte i ärendena 2006/815, 2006/872 och 2006/879
Elisabeth Faxelid (*vårdvetenskap*)
Kristina Gemzell Danielsson (*kvinnosjukdomar*), deltar inte i ärendena 2006/815 och 2006/872 på grund av jäv
Anna Kernell (*barnmedicin*), deltar inte i ärendena 2006/832, 2006/833, 2006/834, 2006/845, 2006/846, 2006/848, 2006/849, 2006/850, 2006/856, 2006/860, 2006/862 och 2006/870
Bernt Lindelöf (*dermatologi*)
Lars Lundell (*kirurgi*), deltar inte i mötet, skriftlig föredragning av ärendena 2006/817, 2006/833 och 2006/834
Christer Paul (*medicin, blodsjukdomar*)
Anette von Rosen (*kirurgi*)
Sven Lindskog (*odontologi*)
Elisabeth Dingertz (*allmänföreträdare*)
Åsa Öckerman (*allmänföreträdare*)

Övriga
Pernilla Asp, *administrativ sekreterare*
Meit Camving, *ordförandens ersättare*

---

§ 1
Ordföranden förklarar mötet öppnat.

§ 2
Ansökningar om etisk granskning av forskningsprojekt, se **Bilaga**

§ 3
Ordföranden meddelar att nästa sammanträde i avdelning 1 äger rum den 20 september 2006.

Olof Forssberg
Ordförande

Pierre Lafolie
Vetenskaplig sekretereare

Adress	Besöksadress	Telefon	Fax	E-post	Hemsida
Box 289<br>171 77 Stockholm	Nobels väg 12 A<br>Solna	08-52480000	08-52486699	kansli@stockholm.epn.se	www.forskningsetikprovning.se

Diarienummer
Föredragande

Utdrag ur protokoll

2006/717-31/1
Elisabeth Faxelid

Sökande: Stockholms läns landsting
Behörig företrädare: Johan Bratt
Projekt: Träning som riktad terapi för patienter med inflammatorisk myopati, myosit – effekter på funktion och muskelvävnad.
Forskare som genomför projektet: Ingrid Lundberg

BESLUT
Nämnden godkänner forskningen på villkor att informationen om behandlingen av känsliga personuppgifter förbättras (se förslag i **underbilaga 1**) och att informerat samtycke avseende biobank inhämtas (se förslag i **underbilaga 2**).

Nämnden erinrar också om att automatiserad behandling av personuppgifter om genetiska anlag som framkommit vid genetisk undersökning skall anmälas för förhandskontroll till Datainspektionen.

Hur man överklagar, se särskild information.

Att utdraget överensstämmer med originalet intygar:

Pernilla Asp, adm sekr /exp 2006-08-29

Karolinska Institutet

2009-01-08

Regionala etikprövningsnämnden i Stockholm
Sid: 1 / 1
2009 -01- 29
Dnr: 2009 / 154 - 32
Handl: [illegible]

Institutionen för Medicin
Enheten för Reumatologi
Ingrid Lundberg, Professor, Överläkare
Reumatologkliniken
Karolinska Universitetssjukhuset, Solna
171 76 Stockholm
08-51776087
ingrid.lundberg@ki.se

Olof Forssberg
Ordförande
Regionala Forskningsetikprövningsnämnden
Stockholm

Ansökan om komplettering till godkänd ansökan till etikprövningsnämnden i Stockholm Dnr:2006/717-31/1
"Träning som riktad terapi vid inflammatorisk muskelsjukdom, myosit"

Vi ansöker härmed om att få utvidga ovanstående singel-centerstudie till multicenterstudie med motiveringen att myosit är en ovanlig sjukdomsgrupp och att en multicenterstudie skulle öka rekryteringshastigheten i projektet.

Vi har hittills inkluderat patienter i god takt som förväntat och testat 16 patienter med polymyosit eller dermatomyosit av förväntade 30 samt sex av förväntade 10 matchade friska kontroller med mikrodialys. 11 patienter har randomiserats i träningsstudien varav 10 fullföljt densamma. Vi har inte haft några problem med mirkodialysundersökningarna under arbete.

Tyvärr har vi under hösten 2008 försinkats i projektet till följd av att utrustningen för max syreupptagningsförmåga vid avdelningen för klinisk fysiologi vid Huddinge har varit ur funktion. Detta är ytterligare ett skäl att inkludera fler centra då det ger möjlighet att utföra max syreupptagningstest vid fler sjukhus.

Samarbete har nu upprättas med reumatologklinikerna vid Akademiska sjukhus och Sahlgrenska sjukhuset, Göteborg, samt med sjukgymnaster vid dessa sjukhus för medverkan i projektet.

Patientinformationerna både den för mikrodialys och träning samt den för muskelbiopsi är modifierad till en multicenterstudie.

Stockholm 2009-01-08

Ingrid Lundberg
Professor, Överläkare

Bilagor:
Intyg från verksamhetschefer
Modifierad patientinformation x2

GODKÄNNES Dat. 2009-02-02

Pierre Lafolie
Regionala etikprövningsnämnden
i Stockholm

Kopians överensstämmelse
med originalet intygas:

Ann-Christin Becker
Administrativ sekreterare
